# Reply to Dr. B. S. Scott, on the Use of “Volasem”

**Published:** 1900-03

**Authors:** G. Lenox Curtis

**Affiliations:** 7 West Fifty-Eighth Street, New York City


### REPLY TO DR. B. S. SCOTT, ON THE USE OF "VOLASEM."

To the Editor:

Sir,—My attention was recently called to Dr. B. S. Scott's letter in the December issue of your Journal, criticising my remarks on volasem before the Northeastern Dental Association, as published in your JOURNAL of April last. Dr. Scott's criticism reflects on my integrity, although he excuses himself by saying,—"It is fair to presume that Dr. Curtis has been misquoted in the report of the Association." He then publishes a letter from Dr. Kelley, which seems to substantiate his views. The Association editor failed to send me a copy of my remarks for correction (as agreed upon), consequently the article was a great injustice to me. My remarks were principally answers to questions put to me by members of the Association as fast as I could answer them. The report of the stenographer is a jumble of all the answers without the questions being given, and I have been misquoted badly in several instances, such as where it states that "Dr. Kelley would not for a minute have thought of performing the operation with cocaine alone;" but what I said of the efficacy of volasem was entirely correct, and I can demonstrate the "positive declaration" I made in regard to it. The facts concerning my authority for referring to Dr. Kelley's use of volasem are these: The patient for whom Dr. Kelley operated has long been an intimate friend of my family and a patient of mine. I have performed oral operations for her under cocaine, with and without volasem. Previous to the use of volasem she always experienced its toxic effect. Knowing this, and being unable to be with the patient at the time of the operation as she desired, I wrote to Dr. Kelley, asking him to use the volasem which I sent him. I quote from his letter in reply to mine. "October 17, 1898. It gives me great pleasure to write you that I did an extensive operation on Mrs. ———'s bladder, urethra, and posterior vaginal wall under cocaine. I gave her, also, the antidote you sent, telling her it was from you. She stood the operation very well indeed, and has been doing splendidly ever since." From a letter from her physician, present at the operation, I quote the following: "The condition of her heart was such that any slight strain could be sufficient cause for sudden death. I therefore feared the effect of ether and also the shock of the operation. Dr. Kelley finally decided he could perform the operation without ether by securing local anæsthesia with cocaine. I observed no unpleasant effects from the cocaine." The patient since her recovery has several times told me that it was decided that only one of the three operations could be done at a time, but when it was found she bore the operation so well that the surgeons decided to finish the others under the one anæsthesia. I quote from a letter from the patient, in which she writes of the operation. "Very soon after swallowing the preferred medicine (volasem) I felt a great relief about my heart and greater courage to endure. After my operation I was not conscious of any deleterious effect from the cocaine."

Any physician who understands the action of cocaine on a heart so weak as this patient should appreciate the value of its antidote as demonstrated here.

G. Lenox Curtis.

7 West Fifty-Eighth Street, New York City.

